# Development of a Rapid Live SARS-CoV-2 Neutralization Assay Based on a qPCR Readout

**DOI:** 10.1128/jcm.00376-22

**Published:** 2022-06-01

**Authors:** Sabine Lichtenegger, Sabine Saiger, Melina Hardt, Susanne Kulnik, Gabriel E. Wagner, Barbara Kleinhappl, Karoline Assig, Andrea Zauner, Michelle Ober, Janine Kimpel, Dorothee von Laer, Kurt Zatloukal, Ivo Steinmetz

**Affiliations:** a Diagnostic & Research Institute of Hygiene, Microbiology and Environmental Medicine, Diagnostic and Research Center for Molecular Biomedicine, Medical University of Graz, Graz, Austria; b Diagnostic & Research Institute of Pathology, Diagnostic and Research Center for Molecular Biomedicine, Medical University of Graz, Graz, Austria; c Institute of Virology, Medical University of Innsbruck, Innsbruck, Austria; Cepheid

**Keywords:** SARS-CoV-2, COVID-19, neutralization assay, neutralizing antibodies, surrogate neutralization assay, cross-neutralization

## Abstract

Measuring SARS-CoV-2 neutralizing antibodies after vaccination or natural infection remains a priority in the ongoing COVID-19 pandemic to determine immunity, especially against newly emerging variants. The gold standard for assessing antibody-mediated immunity against SARS-CoV-2 are cell-based live virus neutralization assays. These assays usually take several days, thereby limiting test capacities and the availability of rapid results. In this study, therefore, we developed a faster live virus assay, which detects neutralizing antibodies through the early measurement of antibody-mediated intracellular virus reduction by SARS-CoV-2 qRT-PCR. In our assay, Vero E6 cells are infected with virus isolates preincubated with patient sera and controls. After 24 h, the intracellular viral load is determined by qRT-PCR using a standard curve to calculate percent neutralization. Utilizing COVID-19 convalescent-phase sera, we show that our novel assay generates results with high sensitivity and specificity as we detected antiviral activity for all tested convalescent-phase sera, but no antiviral activity in prepandemic sera. The assay showed a strong correlation with a conventional virus neutralization assay (r_S_ = 0.8910), a receptor-binding domain ELISA (r_S_ = 0.8485), and a surrogate neutralization assay (r_S_ = 0.8373), proving that quantifying intracellular viral RNA can be used to measure seroneutralization. Our assay can be adapted easily to new variants, as demonstrated by our cross-neutralization experiments. This characteristic is key for rapidly determining immunity against newly emerging variants. Taken together, the novel assay presented here reduces turnaround time significantly while making use of a highly standardized and sensitive SARS-CoV-2 qRT-PCR method as a readout.

## INTRODUCTION

The first case of coronavirus disease 2019 (COVID-19) was reported in December 2019 (1), and the following pandemic has so far resulted in a total of 332,617,707 confirmed cases and 5,551,314 deaths as of January 20, 2022 (2). Two years after the emergence of COVID-19, variants of concern, including Delta and Omicron, are driving new waves of infection worldwide (3), challenging current vaccination strategies (4–6), naturally acquired immunity (7, 8) and antibody-based therapies (9). High levels of neutralizing antibodies (nABs) are recognized as a correlate of protection (10). Serological assays, therefore, remain an essential tool to investigate immune protection in order to validate and optimize vaccines, therapies, and public health measures. These analyses are time critical as rapid adaptation can positively affect viral transmission and clinical management.

Although rapid ELISA-based methods exist (11), they are usually designed for one single virus variant, and most importantly, they only determine the ability of antibodies to bind to viral structures and do not measure functional antiviral responses. Several modified ELISA techniques have been developed recently (12–14) that detect antibodies that interfere in the interaction between the viral receptor binding domain (RBD) and the human host receptor, human angiotensin-converting enzyme-2. Although these assays provide functional information, they are restricted to measuring antibodies against the RBD domain and, consequently, do not include all types of nABs (15–20).

Fast SARS-CoV-2 pseudovirus neutralization assays normally use a vesicular stomatitis virus backbone with their own surface protein replaced by the SARS-CoV-2 spike glycoprotein. As a result, this pseudovirus will bind the human SARS-CoV-2 receptor and viral entry is subsequently measured by a reporter protein (21–23). Nevertheless, the folding, cleavage, and density of spike proteins might differ between pseudovirus particles and the native virus (24–26), which could influence neutralization titers. It should be further noted that pseudovirus-based assays detect nABs that bind to spike protein epitopes and cannot detect antibodies targeting other viral proteins (27).

Live SARS-CoV-2 virus neutralization assays (VNAs), therefore, remain the gold standard for nAB titer assessment as they (i) measure antibodies suppressing infection and (ii) quantify the whole spectrum of nABs (27–29). Most importantly, in contrast to all surrogate assays mentioned, live SARS-CoV-2 assays do not need adaptation to newly emerging variants, reducing the response time to new virus strains. However, a major drawback of standard live SARS-CoV-2 neutralization assays is the incubation time required, which ranges between 3 and 5 days (27, 28, 30, 31). Therefore, these assays do not support a rapid and large-scale screening.

In order to meet the current high demand and circumvent challenges of current fast assays, we developed a rapid live SARS-CoV-2 VNA, which can be performed with all variants without adaptation. The assay relies on the infection of target cells and detection of viral RNA by quantitative RT-PCR (qRT-PCR) after 24 h, massively reducing the time to test result. Using a qRT-PCR based read-out also provides the major benefit of a routinely applied, highly sensitive method usually available in standard diagnostic and research laboratories without the need for special equipment or expertise.

## MATERIALS AND METHODS

### Vero E6 cells.

Vero E6 (ATCC CRL-1586) cells were purchased from the American Type Culture Collection (ATCC; Bethesda, MD) and maintained in minimum essential medium (MEM, ThermoFisher Scientific, Austria) supplemented with 10% fetal calf serum (FCS, ThermoFisher Scientific, Austria) at 37°C with 5% CO_2_.

### Virus strains.

The parental strain was obtained from a clinical case in Germany diagnosed after returning from China (European Virus Archive Global #026V-03883, isolate BetaCoV/Munich/BavPat1/2020). Variants of SARS-CoV-2 were isolated from respiratory swab samples from patients with PCR positive tests at the Medical University of Innsbruck (Beta, B1.351, isolate C24.1, GISAID ID EPI_ISL_1123262) and the Medical University of Graz (Alpha, B.1.1.7). Virus propagation was performed on Vero E6 cells. Cells were infected and supernatant was harvested 72 h after infection, clarified by 0.2 μm filtration, and frozen at −80°C. All work with virus isolates was performed in a Class II Biosafety Cabinet under biosafety level 3 conditions at the Institute of Hygiene, Microbiology and Environmental Medicine, Medical University of Graz.

### Immunoplaque assay.

Immunoplaque assay was used to quantify virus particles in our virus stocks. Plaque-forming units were determined by immunoplaque assay, as described previously (32), with minor modifications. Briefly, confluent monolayers of Vero E6 cells in a 48-well plate were infected with 200 μL virus inoculum and incubated at 37 C for 1 h. Wells were washed twice with 200 μL MEM medium and overlaid with 1.5% carboxymethylcellulose in MEM. Cells were incubated at 37°C in 5% CO_2_ for 3 days before fixing with 4% paraformaldehyde in phosphate-buffered saline (PBS) for 30 min at room temperature. The fixative was removed and wells were washed twice with PBS. Cells were then permeabilized with 0.1% Triton X-100 (Merck, Austria) in PBS for 10 min. The permeabilization buffer was removed and cells were washed with PBS. Cells were incubated with 3% H_2_O_2_ in methanol and subsequently washed with PBS. The primary antibody (anti-nucleoprotein, 40143-R019, SinoBiological) was added and cells incubated for 1 h at room temperature. After washing the cells three times with PBS, a secondary antibody (anti-rabbit, Agilent Technology, Dako, Austria) was added and incubated for 30 min. Cells were washed three times with PBS and incubated with chromogen (Agilent Technology, Dako, Austria) for 1 min. Plaques were observed by microscopy.

### Human sera.

The 69 convalescent-phase sera used in this study (Convalescent Cohort [5003_20]) were obtained from nonhospitalized volunteers (median age = 42 years; range: 21–80) with confirmed COVID-19 infections between March 2020 and January 2021 (11, 33). For cross-neutralization against the Alpha and Beta variants, we exclusively used sera before the emergence of the Alpha and Beta variants in Austria (COVID-19 diagnosis before November 2020). The cohort’s characteristics were published previously by Kral et al. (33). Serum collection and experiments were conducted under protocols reviewed and approved by the Ethical Review Committee of the Medical University of Graz (33-195 ex 20/21). Fourteen days post PCR positivity was selected as the cutoff since the majority of infected individuals show seroconversion at 14 days postinfection (34–37). Twenty non-COVID-19 sera, collected prior to the emergence of COVID-19, were used as negative controls. In order to test for cross-reactivity, a total of 20 prepandemic sera with antibodies against endemic coronaviruses (229E, HKU1, OC43, NL-63), as detected by ProcartaPlex Human Coronavirus Ig Total 11-Plex Panel (Invitrogen, Thermo Fisher Scientific, MA, USA) (38), were tested in the RT-VNA. All sera were heat-inactivated at 56°C for 30 min prior to use.

### ELISA and surrogate neutralization assay.

Sixty-nine convalescent-phase sera from the COVID-19 convalescent cohort (5003_20) were analyzed by the SARS-CoV-2-NeutraLISA (Euroimmun AG, Lübeck, Germany) to quantify immunoglobulin (Ig), which blocks RBD:angiotensin-converting enzyme-2 interaction. Total anti-RBD Ig of the same sera was previously quantified by the Elecsys Anti-SARS-CoV-2S ELISA (Roche Diagnostics GmbH, Mannheim, Germany) during a study, which monitored the immune response of 326 COVID-19 recovered individuals (33). Published Elecsys Anti-SARS-CoV-2S ELISA antibody concentration values of the 69 sera (33) were correlated with neutralization titers of our novel neutralization assay obtained in this study.

### qRT-PCR-based virus neutralization assay.

Twenty-four hours prior to infection, 200 μL of Vero E6 cells (12,000/well) in MEM with 10% FCS were seeded in 96-well plates (Greiner, Austria) and incubated at 37°C and 5% CO_2_. On the following day, heat-inactivated sera were 2-fold serially diluted (1:5–1:20,480) in MEM (without FCS), and 50 μL of the corresponding serum were preincubated with 6 μL of the SARS-CoV-2 strain indicated for 1 h at 37°C. After 1 h, MEM medium was added to reach a final volume of 100 μL. At least four wells of Vero E6 cells were infected with 100 μL of the same serum/virus mixture at a multiplicity of infection (MOI) 0.0025, as determined by immunoplaque assay, and incubated at 37°C in 5% CO_2_ for 1 h. Cells were washed twice with 100 μL MEM and incubated in 200 μL fresh MEM plus FCS at 37°C in 5% CO_2_ for 24 h. After one washing step with 200 μL MEM, cells were lysed with 200 μL eMAG-Puffer (bioMérieux, Austria) for 10 min, which ensures virus inactivation. Therefore, all subsequent steps could be performed under biosafety level 2 conditions. The RNA was isolated with OptiPure viral kit (TANBead, Austria) in a Maelstrom 9600 nucleic acid extractor using 10 μL of the respective sample. The qRT-PCR was performed using the RidaGene SARS-CoV-2 Kit (R-Biopharm, Austria). To determine the viral load in the tested samples, a standard curve was generated. For this genomic, SARS-CoV-2 RNA (VR-1986D, ATCC) at a concentration of 95 ng/mL was used in 1:10 dilutions. The reduction in viral load (%) compared to an immunoglobulin-depleted serum control (serum minus IgA/IgM/IgG human, Merck, Austria) was calculated for each serum concentration. The neutralization titer (NT_90_) was defined as the serum dilution for which 80–99% viral load reduction was observed. For analyzing interassay variability, we used the WHO international standard for anti-SARS-CoV-2 immunoglobulin (39) in the same way as described for human serum (dilution 1:5 −1:10,240).

### Determination of the median tissue culture infectious dose (TCID_50_).

TCID_50_-based assays were conducted, as described previously (28), with minor modifications. SARS-CoV-2 isolates were preincubated with 2-fold serially diluted serum or an immunoglobulin-depleted control at MOI = 0.0025, as determined by immunoplaque assay, for 1 h at 37°C (50 μL serum and 6 μL virus). After 1 h, MEM medium without FCS was added to reach a final volume of 100 μL. An amount of 100 μL of each serum/virus mixture was added to four wells of Vero E6 cells, which were seeded at a cell density of 12,000 cells/well in 96-well plates 24 h prior to infection. Cells were washed twice with 100 μL MEM 1 h after infection and supplied with 200 μL fresh MEM plus FCS for further incubation. After 72 h at 37°C, the cytopathic effect was determined under a light microscope. The NT_50_ reflects the serum dilution at which cytopathic wells are reduced by 50% compared to a 100% viral load control (immunoglobulin-depleted serum).

### Statistical analysis and graphic display.

The association between the different antibody assays was investigated by means of a Spearman correlation. Statistical significance for differences between groups was calculated by Friedman’s test to compare three paired groups. A *P* value of ≤0.05 was considered significant. Analyses were conducted using default parameters in GraphPad Prism (version 9). Graphs were drawn with GraphPad Prism (version 9).

## RESULTS

### The qRT-PCR-based rapid live SARS-CoV-2 neutralization assay correlates with the standard TCID_50_ neutralization assay.

**FIG 1:**
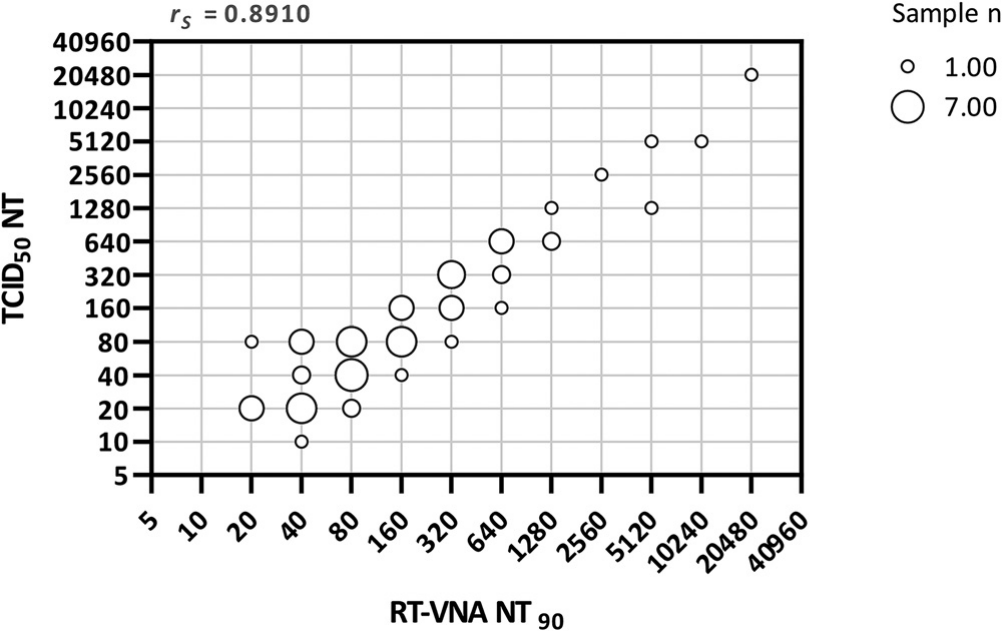
Correlation of the RT-VNA with the TCID_50_-based assay. Bubble chart of RT-VNA NT_90_ (abscissa) versus TCID_50_ NT (ordinate) values. The size of the bubbles indicates the number of sera represented by the data points (varying between 1 and 7). The Spearman correlation coefficient (r_S_) is shown. *n* = 69.

We established a rapid live virus assay with a qRT-PCR-based readout (RT-VNA) applicable for SARS-CoV-2 variants to overcome the challenges of current VNAs. During RT-VNA validation, a NT_90_ defined as a viral load reduction of 80–99% proved to correlate strongly with titers determined by the conventional TCID_50_-based (28) neutralization (Spearman correlation coefficient r_S_ of 0.8910 [*P* < 0.0001]; Fig. 1). Utilizing our assay, we were able to determine neutralization titers for all 69 COVID-19 positive sera (NT_90_ between 20 and 20,480) (Fig. 1). All COVID-19 negative, prepandemic sera (*n* = 40), including 20 positive for other endemic coronaviruses, showed no NT_90_ at 1:5, which is the highest concentration of serum used in the neutralization assay. We determined interassay variability by analyzing the WHO standard for anti-SARS-CoV-2 immunoglobulins (39) in 14 independent experiments, all of which resulted in a neutralization titer of 1,280, demonstrating the high reproducibility of the RT-VNA.

### Correlation of the qRT-PCR-based rapid live SARS-CoV-2 neutralization assay with RBD antibody-binding assays.

**FIG 2:**
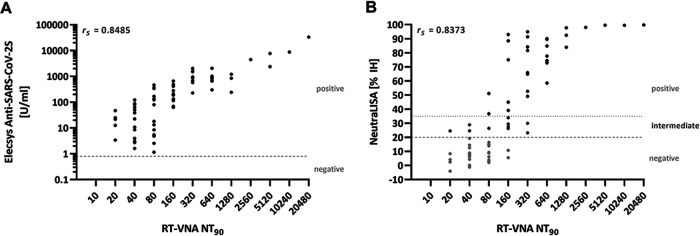
Correlation of the RT-VNA with an antibody-binding assay targeting the receptor-binding domain (Elecsys Anti-SARS-CoV-2S) and a surrogate neutralization assay (NeutraLISA). (A) Chart of RT-VNA NT_90_ (abscissa) versus Elecsys Anti-SARS-CoV-2S (ordinate) values, the latter previously determined in Kral et al. (33). (B) Chart of RT-VNA NT_90_ (abscissa) versus NeutraLISA (ordinate) values. Light gray dots indicate samples rated as negative, dark gray dots samples rated as intermediate, and black dots samples rated as positive in the NeutraLISA. The Spearman correlation coefficient (r_S_) is shown. *n* = 69. U, units; IH, inhibition.

We detected a strong correlation for both the Elecsys Anti-SARS-CoV-2S (r_S_ = 0.8485) and the NeutraLISA (r_S_ = 0.8373) with the RT-VNA (Fig. 2A and B). Similarly, we detected a strong correlation for both the Elecsys Anti-SARS-CoV-2S (r_S_ = 0.8448) and the NeutraLISA (r_S_ = 0.7597) with the TCID_50_-based VNA and also a strong correlation between the Elecsys Anti-SARS-CoV-2S and the NeutraLISA (r_S_ = 0.8231) (Fig. S1 in the supplemental material).

While the RT-VNA, the TCID_50_-based VNA, and the Elecsys Anti-SARS-CoV-2S detected binding antibodies in 69/69 (100%) of convalescent-phase sera (Fig. 2A, Fig. S1A), the NeutraLISA ranked only 43% as positive (30/69) (Fig. 2B), suggesting a lower sensitivity of this assay. Nevertheless, analyzing our pre-pandemic sera collection with the NeutraLISA did not result in a detectable receptor-binding inhibition.

### Quantification of SARS-CoV-2 cross-neutralization by using the RT-VNA.

We analyzed sera collected before the emergence of Alpha (B.1.1.7) and Beta (B.1.351) for their neutralization capacity against the parental strain, and the two variants of concern in order to evaluate whether the RT-VNA is applicable to detect cross-neutralization activity against circulating variants.

**FIG 3:**
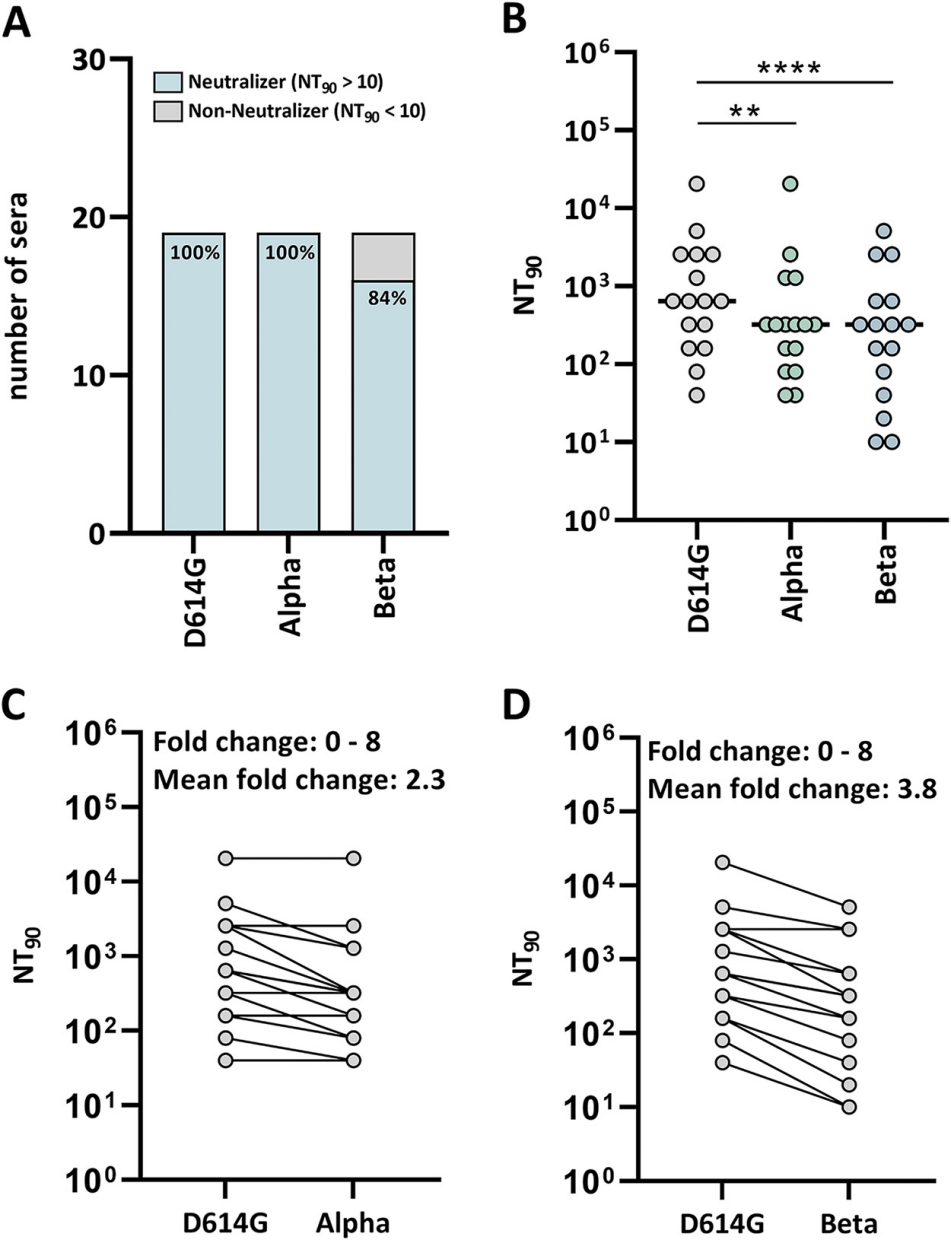
RT-VNA-based detection of D614G (parental SARS-CoV-2 strain), Alpha and Beta neutralization by early wave convalescent-phase sera. (A) Percentage of early wave sera (*n* = 19) tested neutralizing D614G, Alpha and Beta. (B) NT_90_ of early wave convalescent-phase sera (*n* = 16) against Alpha and Beta compared to the early wave strain (D614G). (C) Fold change in the cross-neutralization of Alpha compared to D614G as determined by RT-VNA using early wave convalescent-phase sera (*n* = 16). (D) Fold change in cross-neutralization of Beta compared to D614G as determined by RT-VNA using early wave convalescent-phase sera (*n* = 16). **, *P < *0.01; ****, *P < *0.0001 (Friedman’s test).

The Alpha variant was neutralized by all convalescent-phase sera tested (Fig. 3A). By contrast, 16% of sera showed no neutralization activity against Beta (Fig. 3A). Notably, sera with no neutralization activity against the Beta variant showed only modest activity (NT_90_ = 20–40) against the parental strain. Nevertheless, there was a significant reduction in the antiviral activity of sera against both Alpha and Beta when compared to the parental strain (Fig. 3B). The fold change of the neutralizing capacity (calculated as NT_90_ of the homologous virus/ NT_90_ of the heterologous virus) ranged from 0–8 for Alpha (Fig. 3C) and Beta (Fig. 3D), with a mean fold change of 2.3 for Alpha (Fig. 3C) and 3.8 for Beta (Fig. 3D).

## DISCUSSION

Rapid quantification of the SARS-CoV-2 neutralizing capacity of sera is essential to assess the potential impact of new variants in an immunized population and for timely adaptation of vaccination schemes. It is also needed to identify serum donors for convalescent plasma therapy as well as suitable COVID-19 patients (40).

In the present study, we provide a novel approach for detecting SARS-CoV-2 nABs against currently circulating and future variants. We (i) reduce assay turnaround time massively from several days to 24 h and (ii) use a highly standardized, sensitive, and routinely performed readout by directly quantifying the reduction in intracellular viral RNA, clearly demonstrating that detecting intracellular viral RNA is a valid alternative for determining the neutralization capacity of antibodies. This general test principle was previously demonstrated to be suitable also for other RNA viruses (41). Moreover, Abidi et al. recently reported that RT-PCR can detect antiviral activity in selected sera with high anti-RBD antibody titers in a SARS-CoV-2 neutralization assay (42). However, the authors did not evaluate if their method can be used to determine neutralization titers, since only three different serum dilutions were used, and 13 out of the 19 sera exhibited 100% virus neutralization for all tested concentrations. When analyzing COVID-19 convalescent-phase sera in this study, our RT-VNA generated comparable results to the conventional TCID_50_-based neutralization assay. Our novel RT-VNA is highly sensitive as we were able to determine a neutralization titer for all SARS-CoV-2 convalescent-phase sera, which were positive in the TCID_50_ neutralization assay and the highly sensitive Elecsys Anti-SARS-CoV-2S ELISA (43). The neutralization capacity of convalescent-phase sera in our RT-VNA correlated well with the Elecsys Anti-SARS-CoV-2S ELISA, as has also been demonstrated previously for conventional live VNAs (44). Our assay proved to be highly specific, as we did not determine a NT_90_ titer for SARS-CoV-2 negative sera, including those positive for other endemic coronaviruses (229E, HKU1, OC43, NL-63).

The RT-VNA also correlated well with the NeutraLISA, measuring antibodies that interfere with the spike/angiotensin-converting enzyme-2 interaction. Of note, our results suggested a rather low sensitivity for the NeutraLISA when using the manufacturer’s predefined cutoff values (IH > 20% = positive). In our study, only sera with an NT_90_ of 640 or above were always positive in the NeutraLISA. Nevertheless, a binding inhibition was detected for sera with a low neutralization capacity in the RT-VNA but not for negative pre-pandemic sera. Based on these observations, we suggest a redefinition of the cutoff value of the surrogate neutralization assay. This might be especially relevant for sera collected early after infection or from individuals with a mild disease, the most typical manifestation of COVID-19, as these groups more probably show low antibody levels. Nevertheless, a protective titer still needs to be determined (45).

SARS-CoV-2 variants require immediate attention as they challenge the efficacy of current vaccines and the resistance of recovered COVID-19 patients to reinfection (8, 46). Timely adaptation of current vaccination schemes and public health measures will be essential to contain the spread of these new variants. We exemplarily tested convalescent-phase sera collected before the emergence of Alpha and Beta in Austria for cross-neutralization of the Alpha and Beta variant to demonstrate that our rapid and high-throughput RT-VNA assay is applicable for measuring the neutralization of different virus variants. All convalescent-phase sera tested showed antiviral activity against the Alpha variant in the RT-VNA. This observation is in accordance with previous studies by Rees et al. (47) and Hu et al. (48), who showed, using a pseudovirus assay, that polyclonal antibodies from recovered individuals remain active against this variant. Our observed 2.3-fold reduction in antiviral activity of sera against the Alpha variant when compared to the parental strain, is similar to what was reported by Hu et al. (48). In contrast to the Alpha variant, 16% of convalescent-phase sera did not show any antiviral activity against the Beta variant in this study. Our results obtained with the RT-VNA are in line with studies by Cele et al. (49) and Planas et al. (50), who tested 14 convalescent-phase sera in a live VNA and showed that 17% did not have any cross-neutralization activity. The significant decrease in antiviral activity against the Beta variant compared to the parental strain (3.8-fold reduction as detected in our RT-VNA) is also in accordance with the previous study by Cele et al. (49). Overall, our RT-VNA data on cross-neutralization confirm findings reported previously, which show that early wave sera generally elicit antiviral activity against Alpha but some fail to target the Beta variant.

In conclusion, our live SARS-CoV-2 RT-VNA proved to be a robust tool for analyzing the antiviral activity of sera. We here provide a broadly applicable approach to detect nABs against current and future SARS-CoV-2 variants. Most importantly, the RT-VNA overcomes the major limitation of conventional neutralization assays by massively reducing the turnaround time, thereby, meeting the high demand in the ongoing pandemic.
