# Supplementary material for: Development of a Rapid Live SARS-CoV-2 Neutralization Assay Based on a qPCR Readout
Source: J Clin Microbiol. 2022 Jun 1;60(7):e00376-22. doi: 10.1128/jcm.00376-22 (PMC9297810; doi:10.1128/jcm.00376-22)
Supplement: Supplemental file 1 — Fig. S1. Download jcm.00376-22-s0001.pdf, PDF file, 0.6 MB [file jcm.00376-22-s0001.pdf]

Suppl. Material

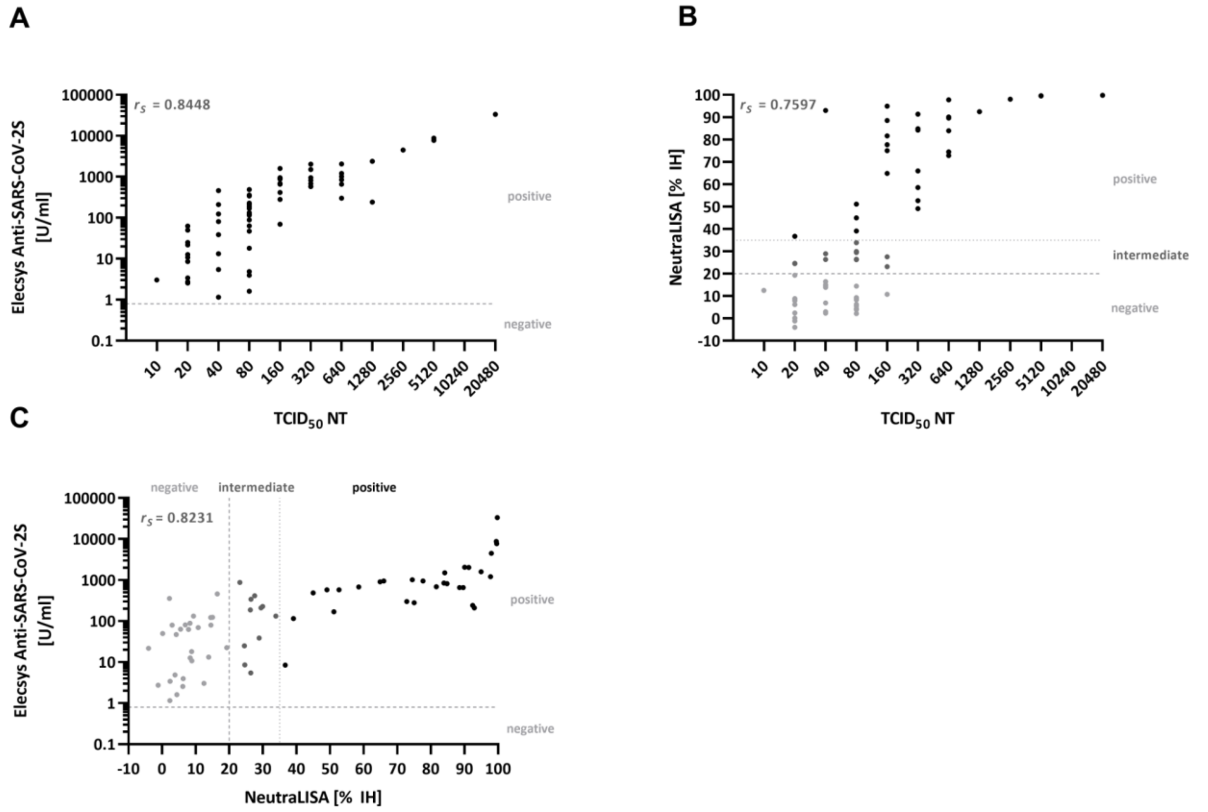

Suppl. Figure 1: Correlation of SARS-CoV-2 neutralization assay ($TCID_{50}$), a surrogate neutralization assay (Euroimmun NeutraLISA) and an antibody-binding assay (Elecsys Anti-SARS-CoV-2S). (A) Chart of $TCID_{50}$ NT (abscissa) versus Elecsys Anti-SARS-CoV-2S (ordinate) values (B) Chart of $TCID_{50}$ NT (abscissa) versus NeutraLISA (ordinate) values (C) Chart of Euroimmun NeutraLISA (abscissa) versus Elecsys Anti-SARS-CoV-2S (ordinate) values. Light grey dots indicate samples rated as negative, dark grey dots samples rated as intermediate and black dots samples rated as positive in the Euroimmun NeutraLISA (B, C). The Spearman correlation coefficient (r_S) is shown. n = 69. U: units. IH: inhibition.
